# Stability and Load-Carrying Capacity of Thin-Walled FRP Composite Z-Profiles under Eccentric Compression

**DOI:** 10.3390/ma13132956

**Published:** 2020-07-02

**Authors:** Hubert Debski, Sylwester Samborski, Patryk Rozylo, Pawel Wysmulski

**Affiliations:** Faculty of Mechanical Engineering, Lublin University of Technology, 36 Nadbystrzycka Str, 20-618 Lublin, Poland; h.debski@pollub.pl (H.D.); s.samborski@pollub.pl (S.S.); p.wysmulski@pollub.pl (P.W.)

**Keywords:** structure stability, composites, limit states, thin-walled composite profiles, FEM

## Abstract

This study investigates the effect of eccentric compressive load on the stability, critical states and load-carrying capacity of thin-walled composite Z-profiles. Short thin-walled columns made of carbon fiber-reinforced plastic composite material fabricated by the autoclave technique are examined. In experimental tests, the thin-walled structures were compressed until a loss of their load-carrying capacity was obtained. The test parameters were measured to describe the structure’s behavior, including the phenomenon of composite material failure. The post-critical load-displacement equilibrium paths and the acoustic emission signal enabling analysis of the composite material condition during the loading process were measured. The scope of the study also included performing numerical simulations by finite element method to solve the problem of non-linear stability and to describe the phenomenon of composite material damage based on the progressive failure model. The obtained numerical results showed a good agreement with the experimental characteristics of real structures. The numerical results are compared with the experimental findings to validate the developed numerical model.

## 1. Introduction

Nowadays, thin-walled load-carrying structures are made of modern construction materials, such as polymer matrix composites reinforced with continuous fibers-FRPs. To ensure their high strength and stiffness, these elements are fabricated with the autoclaving technique. A classic example of load-carrying FRP composite structural components is thin-walled profiles with open or closed sections that are used to stiffen fuselages of airplanes [1,2]. These profiles are high load-carrying capacity structural components subjected primarily to axial or bending loads [3,4,5,6]. The description of problems recently considered for modern composite materials can be found, for instance, in [7,8,9], where the authors present mainly experimental research on the failure of the composite material. The studies presented in the abovementioned works are described using the example of thin-walled structures made of glass fiber-reinforced plastic and fiber metal laminate materials. An important stage of the loss of load-carrying capacity analysis is the analysis of the stability of the structure in the context of the use of acoustic emission [10]. The issues raised in this paper reflect the current area of interest of modern materials designers and researchers well. The main limitation in the design of thin-walled structures is the occurrence of a global or local loss of stability of the entire stiffening profile or its particular walls, which can significantly change the nature of the load-carrying capacity of these structural components.

There are several problems with FRP composites that must be considered when introducing them to various load-bearing structures, such as them exceeding metals or other engineering materials due to their high strength to mass ratio [10]. One of the basic weakness of the FRP laminates is their susceptibility to different forms of damage, among which delamination predominates [9]. This form of material deterioration is understood as interlaminar cleavage that can be studied within the framework of Fracture Mechanics, as 2D or 3D crack initiation and propagation [11,12,13]. This demands the determination of fracture toughness with standardized procedures, providing globally the three classical fracture modes [14,15,16,17], which may be a demanding task when atypical layups come into play, for example those evoking elastic couplings [18]. In any case, damage detection, monitoring and identification is possible with contemporary experimental methods supported by appropriate modelling techniques, such us the Finite Element Method (FEM) and analytical solutions. Particularly, the detection of internal defects, such as delamination, can be performed with the Acoustic Emission (AE) method [10]. This method is based on vibrations induced in the material space due to the elastic energy release along with defects’ formation. Note that the contemporary AE equipment also enables damage identification thanks to the advanced software tools for signal analysis such as the Fast Fourier Transform (FFT) [10]. Damage in the FRP composite structures can also be identified with other vibrational techniques that work well both in the experiment and in the FEM modeling [19,20,21,22]; they allow for friction or thermal effects and give an insight into the interlaminar forces/stress state in the dynamic regime, which is inevitable even in outwardly static problems (in a global sense), as the propagation of delamination can be piecewise rapid or even unstable. On the other hand, the abovementioned complicated studies omit the detailed modeling of the internal structure (resin, fibers, voids, etc.) [23], which can have a significant effect on fracture resistance [24].

The phenomenon of thin-walled profile buckling is highly undesired because it may occur within the range of permissible operating loads. This problem was raised, among others, in [25,26,27,28,29,30,31], where the authors showed that a structure with local buckling of its elements can still be used, provided that the mode of stability loss is elastic buckling and that the post-critical equilibrium path is stable. This observation has also been confirmed by the author’s previous studies [32,33,34,35] devoted to the loss of stability of compressed thin-walled composite profiles, the results of which have shown that, in spite of buckling, thin-walled profiles retain a considerable reserve of load-carrying capacity allowing for the safe bearing of loads in the post-critical range.

Another undesired phenomenon regarding thin-walled profiles is the occurrence of various types of inaccuracies that may significantly affect the performance of such structures. These inaccuracies include geometric imperfections resulting from the manufacturing process, whose impact on the behavior of compressed laminated beams and I-profiles is described, among others, in [36,37]. A different type of inaccuracies is those related to asymmetrical boundary conditions; this problem is shown in [38], where their significant impact on the critical and failure loads of compressed C-profiles was demonstrated. The problem of compressive load eccentricity is of vital importance because—depending on the direction of the load eccentricity relative to the center of gravity of the profile—it can lead to significant changes in the nature of the stress distribution of the thin-walled element [39]. The effect of eccentric compression on the behavior of composite plates and profiles was already investigated in [40,41,42], where the authors presented the results of experimental and numerical tests confirming that load eccentricity reduced the structure’s strength, leading to a loss of its structural stability and load-carrying capacity. The research in the abovementioned works was carried out on thin-walled structures with open cross-sections.

Recently, the latest scientific research has shown innovative solutions for analyses on thin-walled composite materials in terms of crashworthiness [43], where the process of dynamic crushing on thin-walled structures which had different cross-sections was presented.

Given the significance of the above problems, an in-depth study has been undertaken to investigate the impact of the eccentric compression on the stability and load-carrying capacity of thin-walled FRP composite Z-profiles. The study described in this paper covers a numerical and an experimental analysis of the non-linear stability and load-carrying capacity of carbon-epoxy composite profiles under compression, with real-time damage detection and its identification. The analyzed Z-profiles are subjected to a non-axial compression in two mutually perpendicular directions in the full load range until failure. A FEM numerical model is developed and then validated by experimental results.

## 2. The Object of Study 

**Figure 1:**
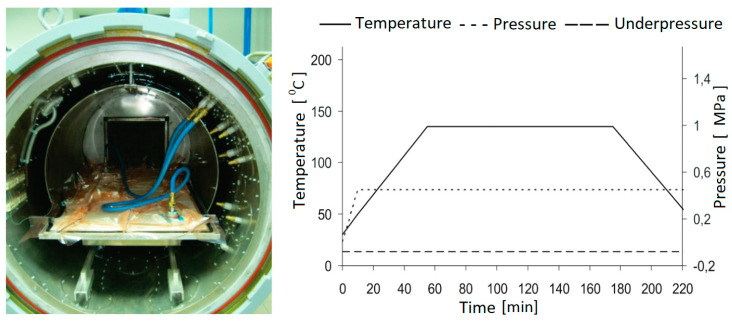
Manufacturing process.

The study investigated short thin-walled Z-profiles made of prepreg GFRP semi-finished products fabricated by the autoclave technique, which made it possible to obtain a composite with a percentage of fibers in the range of 55–60%. The manufacturing process included the preparation of a hermetic vacuum package in a special clean room, as shown in Figure 1. The applied manufacturing process allowed for the complete automation of the manufacturing process, allowing us to obtain specimens with high repeatability, high strength properties and low porosity.

**Figure 2:**
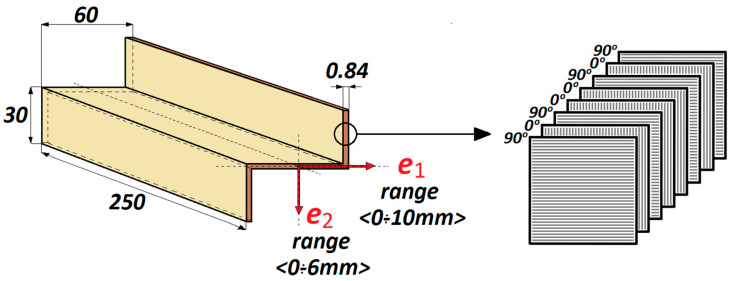
Geometric dimensions of the analyzed Z-profile.

The laminate was symmetric and had the stacking sequence [90/0/90/0] s. The Z-profiles had the following geometric dimensions: w = 60 mm, h = 30 mm, profile length l = 250 mm and wall thickness t = 0.84 mm. Eccentric load was applied in two mutually perpendicular directions (Figure 2): parallel to the web of the Z-profile (direction *e*_1_) and perpendicular to the profile’s web (direction *e*_2_), in the following ranges: *e*_1_<0–10 mm> and *e*_2_<0–6 mm>. The geometric dimensions of the analyzed Z-profile and the stacking sequence of the laminate plies are shown in Figure 2.

## 3. Experimental Methods 

**Figure 3:**
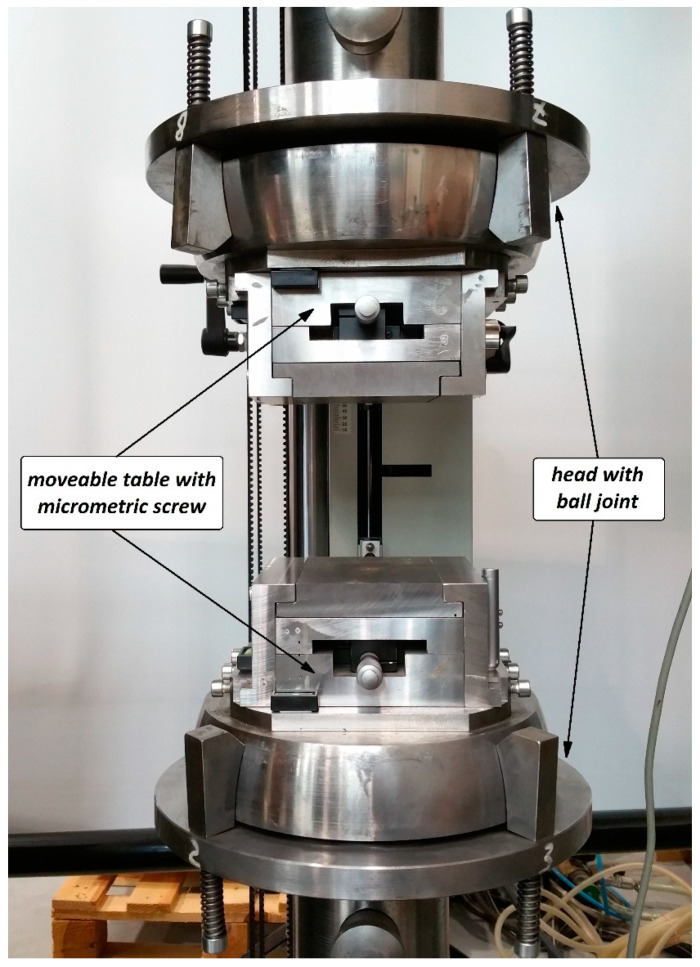
Experimental setup.

In the experimental tests, the Z-profiles were subjected to a non-axial compression in the full load range until the loss of their load-carrying capacity. The experiments were performed at a room temperature of 20 °C on a universal testing machine (Zwick 100, ZwickRoell GmbH & Co. KG, Ulm, Germany). The load was applied by the upper crosshead moving with a constant speed of 2 mm/min. As part of the conducted research, the following were measured: the loading force, the profile displacement and the acoustic emission signal. The measurement of the acoustic emission signal using a piezoelectric sensor enabled the ongoing assessment of the condition of the composite material during loading. Figure 3 shows the experimental setup with a mounted sample.

During the tests, the specimen was simply supported on the surface of moveable tables provided with a micrometric screw to move the specimen relative to the axis of the testing machine, and thus to ensure the precise application of the eccentric load. In addition, the application of the load eccentricity was monitored with electronic sensors, as shown in Figure 3. The post-critical characteristics of the structure’s behavior were determined as a load vs. profile displacement relationship for the full operating range of the structure. These characteristics were then compared with the results of the acoustic emission signal measurement in order to identify potential damage of the composite structure. Two characteristic load values were determined: the composite damage initiation load, *P*_d_/EXP, and the failure load, *P*_f_/EXP, at which the structure loses its load-carrying capacity. The experimentally determined characteristics relating to the real structure’s behavior (four actual profiles in the context of the failure were tested: for 0 mm, 6 mm in the *e*_2_ direction, as well as 5 and 10 mm in the *e*_1_ direction) and its characteristic load values served as a basis for validating the numerical models.

## 4. Numerical Analysis 

The problem of the stability and load-carrying capacity of the compressed composite columns was solved numerically via the finite element method. The numerical calculations were made in two stages. The first stage of the calculations involved solving the problem of structural stability loss, constituting a linear Eigen problem of the structure without initial geometric imperfections. As a result, it was possible to determine the lowest mode of structural stability loss and the corresponding critical load using the minimum total potential energy principle [44].

The second stage of the analysis involved solving a nonlinear stability problem. The numerical calculations were performed via the Newton–Raphson incremental–iterative method. The analyzed model had an initial geometric imperfection corresponding to the lowest mode of stability loss obtained in the linear analysis. In the analyzed case, the amplitude of initial geometric imperfections was *w*_0_ = 0.02 mm. This value was determined in the test calculations carried out for different values of initial geometric imperfections; for the applied value of imperfection, the post-critical characteristics of the modelled structure were the closest to the characteristics of the real structure. The numerical analysis was carried out using the commercial FEM-based program ABAQUS® (Abaqus 2019, Dassault Systemes Simulia Corporation, Velizy Villacoublay, France).

The damage of the composite material was analyzed using the fundamental damage model [45], according to which the relationship between the effective stress σ^^^ and the nominal stress σ is expressed by the damage operator M having the form:

(1) $$\hat{\sigma }=M\sigma =\left[\begin{array}{ccc} \frac{1}{1-d_{f}} & 0 & 0\\ 0 & \frac{1}{1-d_{m}} & 0\\ 0 & 0 & \frac{1}{1-d_{s}} \end{array}\right]\sigma$$

where *d*_f_, *d*_m_ and *d*_s_ are the damage variables for the fiber, matrix and shear failure modes, respectively. Prior to any damage initiation and evolution, the damage operator M is equal to the identity matrix, hence σ^^^ = σ. A similar approach to anisotropic damage description was also presented in works for a quasi-static loading regime [46].

Damage of the compressed composite columns was examined with respect to the identification of the point of first ply damage and the description of a loss of the load-carrying capacity of the entire structure. To this end, the load causing first ply damage (*P*_d_/FEM) and the failure load (*P*_f_/FEM) were determined. The composite damage initiation was described based on the Hashin theory [47,48], according to four independent damage initiation criteria: fiber tension, fiber compression, matrix tension and matrix compression. Individual initiation criteria were described by the following relationships:

(2) $$\mathrm{fiber}\;\mathrm{tension}\;\left(\sigma_{11}\geq 0\right):\;F_{f}^{T}={\left(\frac{{\hat{\sigma }}_{11}}{X^{T}}\right)}^{2}+\alpha {\left(\frac{{\hat{\tau }}_{12}}{2S}\right)}^{2}$$

(3) $$\mathrm{fiber}\;\mathrm{compression}\;\left(\sigma_{11}<0\right):\;F_{f}^{C}={\left(\frac{{\hat{\sigma }}_{11}}{X^{C}}\right)}^{2}$$

(4) $$\mathrm{matrix}\;\mathrm{tension}\;\left(\sigma_{22}\geq 0\right):\;F_{m}^{T}={\left(\frac{{\hat{\sigma }}_{22}}{Y^{T}}\right)}^{2}+{\left(\frac{{\hat{\tau }}_{12}}{2S}\right)}^{2}$$

(5) $$\mathrm{matrix}\;\mathrm{compression}\;\left(\sigma_{22}<0\right):\;F_{m}^{C}={\left(\frac{{\hat{\sigma }}_{22}}{2S}\right)}^{2}+\left[{\left(\frac{Y^{C}}{2S}\right)}^{2}-1\right]\frac{{\hat{\sigma }}_{22}}{Y^{C}}+{\left(\frac{{\hat{\tau }}_{12}}{2S}\right)}^{2}$$

where *X*^T^ and *X*^C^ are the longitudinal tensile and compressive strengths, respectively; *Y*^T^ and *Y*^C^ are the transverse tensile and compressive strengths; and *S*^L^ and *S*^T^ are the longitudinal and transverse shear strengths, respectively. The coefficient α in Equation (2) determines the contribution of the shear stress to the tensile fiber damage initiation criterion. The employed calculation procedure enables an independent assessment of the composite fiber and matrix damage.

When any of the damage initiation criteria are exceeded, further loading of the structure causes the damage to progress. This mechanism is described by a progressive damage model that is a generalization of the Camanho and Davila theory [49] formulated for cohesive elements. The above model allows the description of composite material damage progression leading to a complete loss of the material’s rigidity. The composite matrix stiffness can be expressed as:

(6) $$\left\{\begin{array}{c} \sigma_{11}\\ \sigma_{22}\\ \tau_{12} \end{array}\right\}=\frac{1}{D}\left[\begin{array}{ccc} \left(1-d_{f}\right)E_{1} & \left(1-d_{f}\right)\left(1-d_{m}\right)v_{21}E_{1} & 0\\ \left(1-d_{f}\right)\left(1-d_{m}\right)v_{12}E_{2} & \left(1-d_{m}\right)E_{2} & 0\\ 0 & 0 & D\left(1-d_{s}\right)G_{12} \end{array}\right]\left\{\begin{array}{c} \varepsilon_{11}\\ \varepsilon_{22}\\ \gamma_{12} \end{array}\right\}$$

where σ*_ij_* are the stresses in the *ij* direction, c*_ij_* are the stiffness coefficients, ε*_ij_* are the strains, τ*_ij_* are the shear stresses, γ*_ij_* are the shear strains and D is expressed as:

(7) $$D=1-v_{12}v_{21}$$

The adopted progressive damage model includes five independent composite material damage criteria: fiber compression, fiber tension, matrix compression, matrix tension and shear failure. As a result, it allows for a complex analysis of composite material damage at the moment of a complete loss of the structure’s load-carrying capacity.

**Figure 4:**
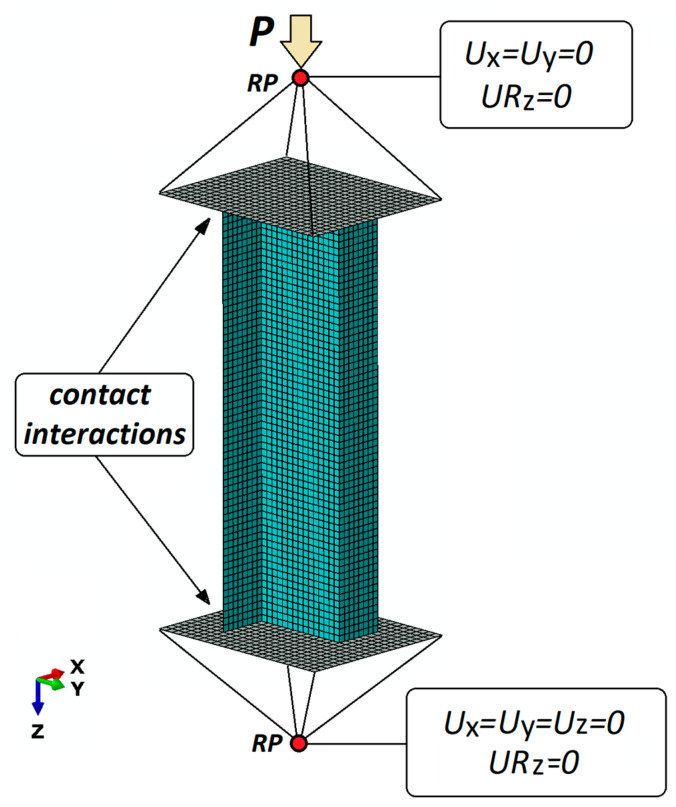
Numerical model of the analyzed structure.

Figure 4 shows the developed numerical model of the analyzed composite structure under compression. The model consists of a Z-profile, rigid plates supporting the column ends and reference points that are ascribed to the boundary conditions of the model and reflect the ball joints of the mounting heads.

The Z-profile under study was discretized using eight-node shell finite elements with six degrees of freedom in every node. Known as S8R, this type of finite element has a second order shape function and reduced integration. The laminate structure was defined with respect to the finite element thickness, with individual laminate plies separated. The modelled material was assigned the properties of an orthotropic material in plane stress, assuming the experimentally determined (the properties of the composite material determined based on ISO standards: static tensile test—ISO 527, compression test—ISO 14126 and shear test—ISO 14129) mechanical and boundary properties of the composite material, as listed in Table 1 [50]. The values of the fracture energy and regularization were found in the bibliography [51].

According to the developed numerical model, the profile is simply supported on rigid plates. The contact conditions between the edges of the profile and the plate surface reflect the frictional contact in the normal and tangential direction described by the coefficient of friction equal to 1. The boundary conditions of the numerical model are defined at reference points, RP, whose locations correspond to the centers of gravity of the ball joints of the mounting heads mounted on the testing machine. The reference points do not take into account the coefficient of friction that is typical of ball joints. The reference points and the plates were described by rigid contact relations to connect all kinematic degrees of freedom. The loading of the model is modelled as a concentrated compressive force applied to the upper reference point.

## 5. Results

### 5.1. Composite Material Damage Initiation

**Figure 5:**
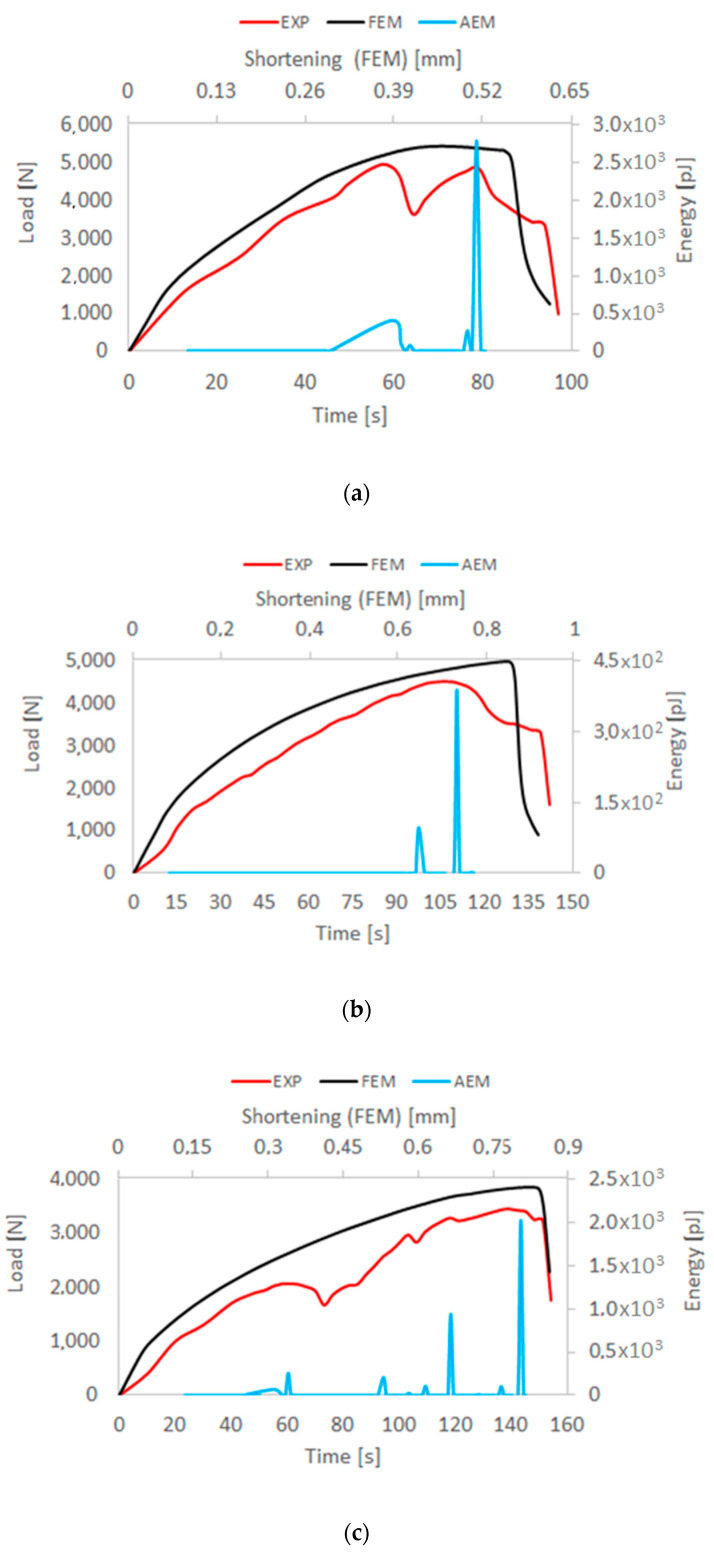
Comparison of the experimental and numerical characteristics of the structure (load vs. displacement): (**a**) axial compression; (**b**) eccentricity *e*_1_ = 10 mm; (**c**) eccentricity *e*_2_ = 6 mm.

The loss of stability of the compressed Z-profiles is manifested as the local buckling of the walls and web of the structure, assuming the form of three half-waves in the longitudinal direction of the column under axial compression. It can be observed that the application of the load eccentricity *e*_1_ does not change the buckling mode, whereas for the maximum value of the load eccentricity *e*_2_ the buckling mode changes from three to four half-waves. With further loading of the structure, the deformations corresponding to the lowest buckling modes increase. The post-critical load vs. deflection characteristics (*P*-*u*_z_) remain stable, which confirms that the structure can carry the compressive load in spite of buckling [52,53,54,55,56,57], as shown in Figure 5. The experimental (testing machine) and numerical (FEM) characteristics are compared with the results of the AE signal energy—the results are plotted for the axial compression case and for extreme values of the load eccentricity applied in the two perpendicular directions *e*_1_ and *e*_2_.

**Figure 6:**
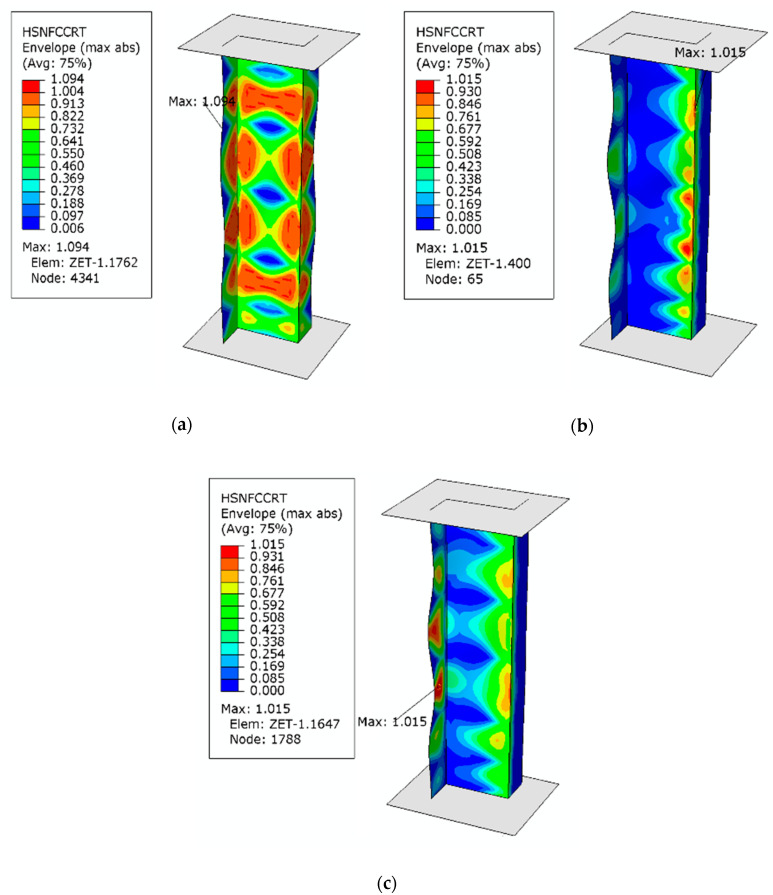
HSNFCCRT of the analyzed composite structures: (**a**) axial compression; (**b**) *e*_1_ = 10 mm; (**c**) *e*_2_ = 6 mm.

The high agreement between the numerical and experimental results confirms the adequacy of the developed numerical model and thus provides a basis for conducting an in-depth analysis of the structure’s behavior, including the assessment of the damage phase. The initiation of the composite material damage in the real structure was identified on the basis of the first clear increase in the acoustic emission signal. According to the employed method, it was assumed that the first clear increase in the value of the acoustic signal would correspond to the damage initiation (related to the first ply failure) of the composite material. In effect, it was possible to determine the composite material damage initiation load *P*_d_/EXP. The value of the composite damage initiation load was compared with the load *P*_d_/FEM (first ply damage load) that was determined in the numerical calculations when the damage initiation criterion reached the value of 1. Figure 6 shows the maps of the compressive fiber damage variable for the axial compression case and extreme values of the load eccentricities *e*_1_ and *e*_2_.

The numerical results make it possible to locate the areas in which the composite material damage initiates. An analysis of these results reveals that in all tested cases the composite material damage initiates in Ply 2 and 7, starting with compressive fiber damage (HSNFCCRT variable).

**Figure 7:**
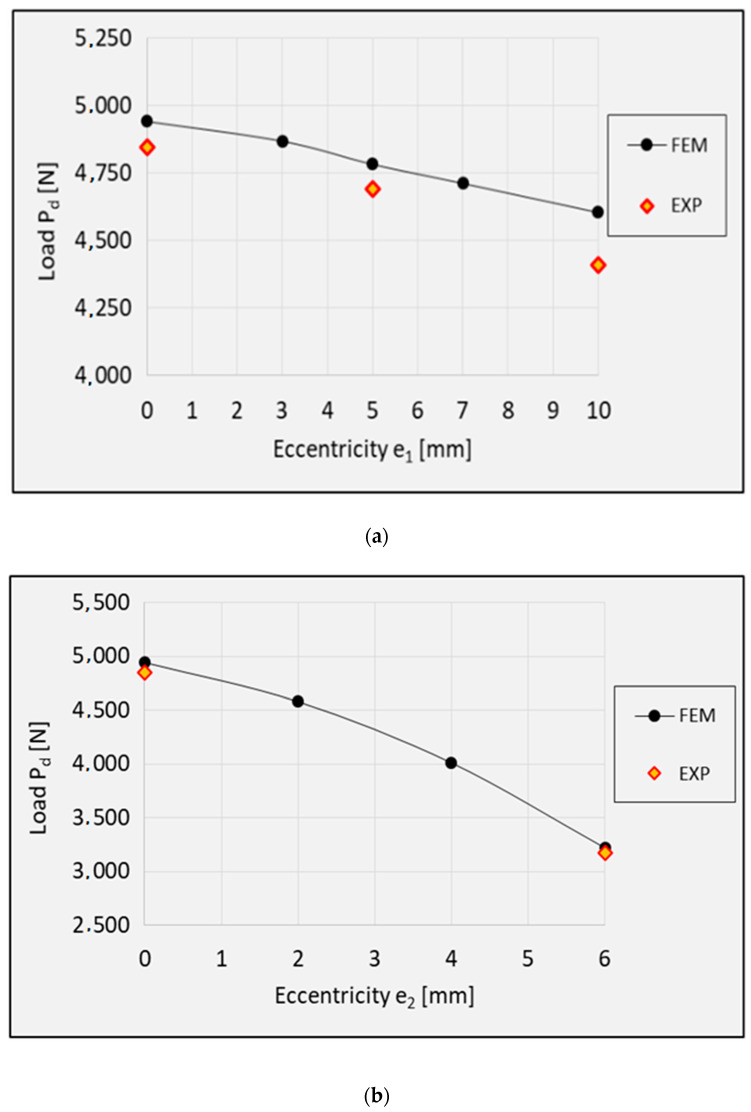
Composite material damage initiation load P_d_: (**a**) eccentricity *e*_1_; (**b**). eccentricity *e*_2_.

Figure 7 shows the composite material damage initiation loads depending on the value of the load eccentricity. As a result, it is possible to determine the effect of the load eccentricity on the composite material damage initiation. The experimental results are compared with the results of the numerical calculations. The maximum differences between the experimental and numerical results do not exceed 4.2% (Table 2).

The obtained values of the composite material damage initiation load are listed in Table 2. 

The results demonstrate that, irrespective of the eccentricity direction, an increase in the eccentricity causes a monotonic decrease in the composite material damage initiation load. This means that the application of eccentric loading leads to a faster degradation of the composite material, which is undesired due to strength reasons. 

### 5.2. Loss of The Load-Carrying Capacity–Limit States

**Figure 8:**
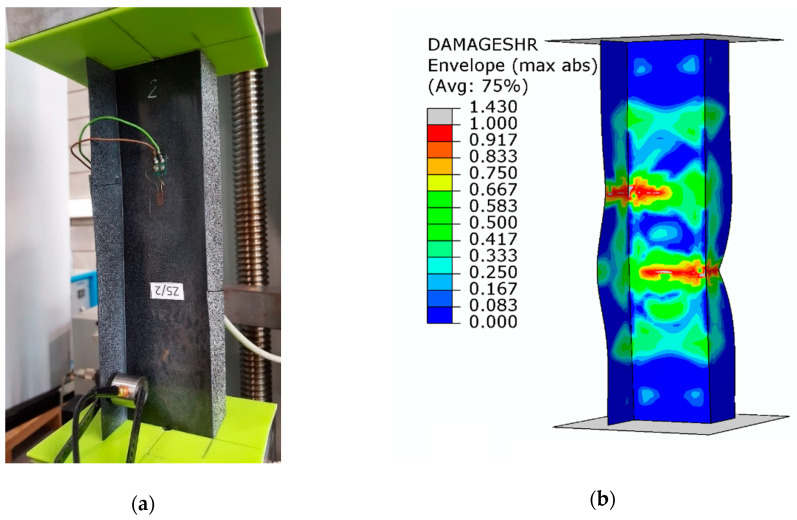
Buckling mode of the structure under axial compression: (**a**) experimental; (**b**) numerical.

**Figure 9:**
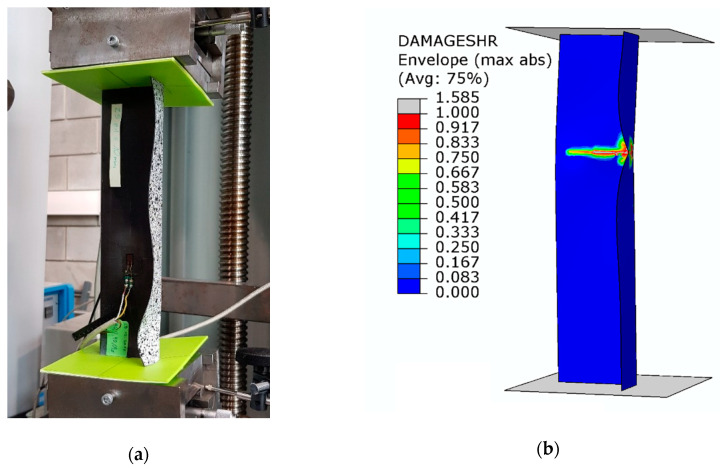
Buckling mode of the structure under the load eccentricity *e*_1_ = 10 mm: (**a**) experimental; (**b**) numerical.

**Figure 10:**
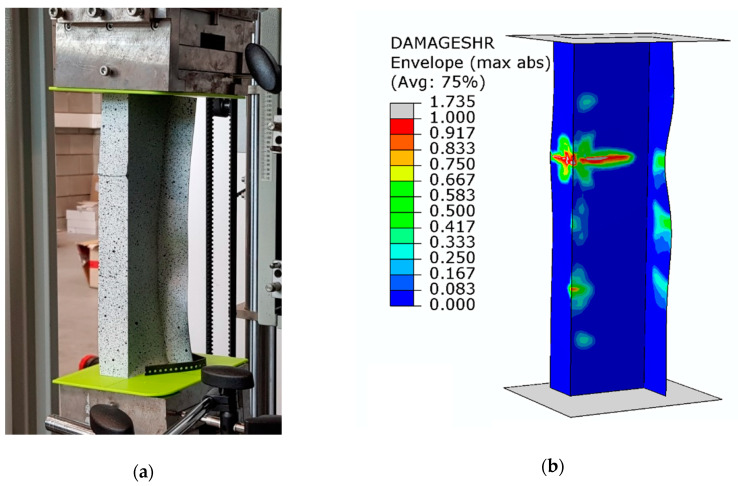
Buckling mode of the structure under the load eccentricity *e*_2_ = 6 mm: (**a**) experimental; (**b**) numerical.

Despite the composite material damage initiation, the structures retain their load-carrying capacity and can still carry compressive loads. This is evidenced by a further increase in the force after exceeding the load *P*_d_, as shown by the post-critical characteristics of the structure in Figure 5. In the numerical analysis, the evolution of the initiated damage was modelled as a progressive degradation of the composite material stiffness as determined by the energy criterion. The progressive degradation of the stiffness of the individual variables of the composite material leads to a complete loss of the load-carrying capacity of the structures, that is, the structures are unable to carry the compressive load any further. The value of the load at which the structure loses its load-carrying capacity is expressed as a failure load, *P*_f_. Figure 8, Figure 9 and Figure 10 show the buckling modes of the structure under axial compression and extreme values of the load eccentricity along *e*_1_ and *e*_2_.

**Figure 11:**
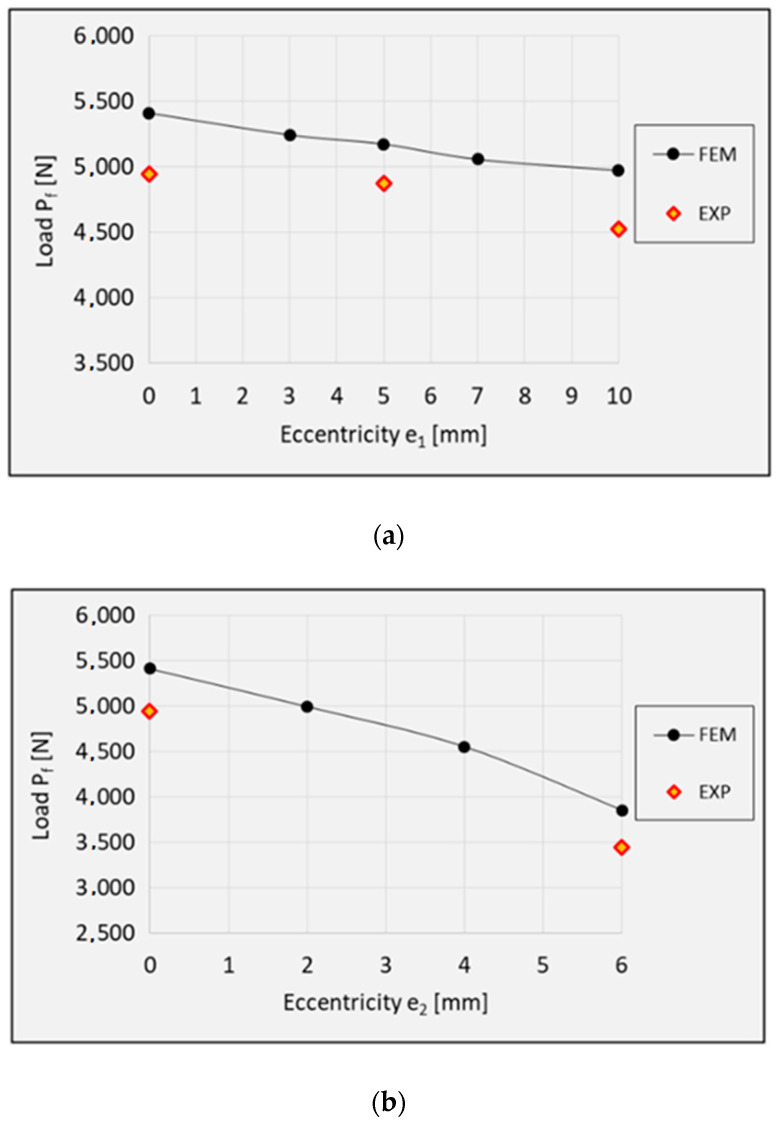
Failure load: (**a**) eccentricity *e*_1_; (**b**) eccentricity *e*_2_.

The results show that the real structures and the numerical models behave in a very similar manner. This concerns both the nature of the obtained post-critical characteristics (Figure 5) and the modes of structural damage (Figure 8, Figure 9 and Figure 10), which confirms the quantitative and qualitative agreement between the experimental and the numerical results. An analysis of the numerical results reveals that the compressed columns lose their load-carrying capacity when the failure criteria are satisfied by all variables describing the composite material. The exception to this is the fiber tensile damage variable (DAMAGEFT), which occurs at a slightly later stage of the loading process, after the loss of the load-carrying capacity by the structure. A quantitative analysis of the results makes it possible to determine the effect of eccentricity on the failure load *P*_f_. A comparison of the numerical and experimental results for selected values of the load eccentricity (due to the limited number of test samples) is shown in Figure 11.

Table 3 lists the obtained failure loads depending on the direction of the eccentricity.

The application of eccentric loading causes a monotonic decrease in the failure load. It should however be emphasized that the compressed Z-profiles are more sensitive to the eccentricity along the direction *e*_2_, perpendicular to the web of the profile. The application of eccentricity along *e*_1_ in the range of 0–10 mm caused a decrease in the failure load; in comparison to the axial compression case, the failure load decreased by 8.1% (numerical calculations) and 8.5% (experimental tests). As for the load eccentricity *e*_2_, despite the significantly lower maximum eccentricity value (0–6 mm), the failure load decreased by 28.8% (numerical calculations) and 30.4% (experimental tests). This results from a higher structural stiffness along the web of the Z-profile with rigid edges.

## 6. Conclusions

The study proposed a numerical and experimental research methodology enabling the determination of the impact of eccentric compression on the stability and load-carrying capacity of composite Z-profiles.

The results of the study demonstrated that the load eccentricity along two perpendicular directions (parallel–*e*_1_ and perpendicular–*e*_2_ to the web of the Z-profile) had a negative effect on the analyzed structures. Of the two, the least favorable results were obtained for the load eccentricity perpendicular to the web of the profile. Despite the low value of the eccentricity (*e*_2_ = 6 mm), the failure load decreased by 28.8% (numerical analysis) and 30.4% (experimental tests) in comparison to the axial compression case. This situation was caused by the change in the distribution of stresses and strains in the thin-walled profile when compared to the structure under axial compression.

The proposed numerical solution, involving the use of a numerical analysis via a progressive model of composite material damage, made it possible to identify the moment and the point of initiation of damage in the composite structure and to assess the state of damage at the moment of load-carrying capacity loss by the structure. It was found that the damage of the compressed composite structure initiated in Ply 2 and 7 and manifested as compressive fiber damage. Once initiated, the damage process evolved, resulting in a complex mechanism of failure, which meant that the structure lost its load-carrying capacity when all composite damage initiation criteria were satisfied in the area of damage. The numerical results were in agreement with the experimental results obtained for the real composite structures, which confirms both the correctness of the employed research methods and the adequacy of the developed numerical models.

## Figures and Tables

**Table 1:** Mechanical and strength properties of the composite material.

Tensile Strength *F*_TU_ [MPa]		Tensile Modulus *E*_T_ [MPa]		Poisson’s Ratio ν_12_	Shear Strength *F*_SU_ [MPa]	Shear Modulus *G* [MPa]	Compression Strength *F*_CU_ [MPa]	
0°	90°	E_1_ (0°)	E_2_ (90°)	0°	±45°	±45°	0°	90°
2220.7	49	143,528.5	5826.3	0.36	83.5	3845.5	641	114
Fracture energy *G*_1t_ Fiber tension [N/mm]		Fracture energy *G*_1c_ fiber comp. [N/mm]		Fracture energy *G*_2t_ matrix crack. [N/mm]	Fracture energy *G*_2c_ matrix crush. [N/mm]		Viscosity coefficients *η*_1t_, *η*_1c_, *η*_2t_, *η*_2c_ [-]	
133		10		0.5	1.6		0.0005	

**Table 2:** Composite material damage initiation load *P*_d_ versus eccentricity.

Eccentricity [mm]	Direction *e*_1_						Direction *e*_2_		
	0	3	5	7	10	0	2	4	6
Numerical [N]	4941	4867	4782	4711	4603	4941	4578	4010	3219
Experimental [N]	4847	-	4691	-	4408	4847	-	-	3172
Difference [%]	1.9	-	1.9	-	4.2	1.9	-	-	1.5

**Table 3:** Composite material damage initiation load *P*_d_ versus eccentricity.

Eccentricity [mm]	Direction *e*_1_						Direction *e*_2_		
	0	3	5	7	10	0	2	4	6
Numerical [N]	5408	5241	5171	5054	4969	5408	4992	4551	3852
Experimental [N]	4941	-	4872	-	4521	4941	-	-	3440
Difference [%]	8.6	-	5.7	-	9.0	8.6	-	-	10.7
